# Visual Attention in Flies—Dopamine in the Mushroom Bodies Mediates the After-Effect of Cueing

**DOI:** 10.1371/journal.pone.0161412

**Published:** 2016-08-29

**Authors:** Sebastian Koenig, Reinhard Wolf, Martin Heisenberg

**Affiliations:** Rudolf Virchow Center for Experimental Biomedicine, University of Würzburg, Joseph-Schneider-Straße 2, 97080, Würzburg, Germany; Biomedical Sciences Research Center Alexander Fleming, GREECE

## Abstract

Visual environments may simultaneously comprise stimuli of different significance. Often such stimuli require incompatible responses. Selective visual attention allows an animal to respond exclusively to the stimuli at a certain location in the visual field. In the process of establishing its focus of attention the animal can be influenced by external cues. Here we characterize the behavioral properties and neural mechanism of cueing in the fly *Drosophila melanogaster*. A cue can be attractive, repulsive or ineffective depending upon (e.g.) its visual properties and location in the visual field. Dopamine signaling in the brain is required to maintain the effect of cueing once the cue has disappeared. Raising or lowering dopamine at the synapse abolishes this after-effect. Specifically, dopamine is necessary and sufficient in the αβ-lobes of the mushroom bodies. Evidence is provided for an involvement of the αβ_posterior_ Kenyon cells.

## Introduction

Flies (*Drosophila melanogaster*) like other animals and humans can restrict their visual responses to parts of the visual field [1–6]. This property of vision is called selective visual attention (SVA). More appropriately it should be called 'spatially selective visual attention' to distinguish it from attention to novelty or other visual features such as colors, patterns or motion (for a review see [7]). SVA refers exclusively to spatial selectivity. The fly is assumed to have a focus of attention (FoA) which it can spontaneously shift to particular areas of its visual field and to be more likely to respond to visual stimuli occurring in this region than elsewhere.

In flies SVA has been studied mainly in stationary flight of tethered animals. Under these highly restricted experimental conditions visual stimuli can be presented in defined regions of the fly's visual field and the fly shows by its intended turning (yaw-torque) to which of the stimuli it responds. For instance, one can present two black stripes at symmetrical positions in the fronto-lateral visual field of the fly and move them suddenly at the same time from front to back as seen by the fly [5]. The fly responds to each of the stripes in about 1/3 of the trials, presumably to the one closest to its FoA. In 1/3 of the trials the fly does not respond at all. Whether this implies that neither stripe happens to be close enough to the FoA is not known.

Using this test we have recently shown that the fly keeps the FoA for several seconds at the location to where it has endogenously shifted it [6]. In humans this endogenously driven process is called a top-down modulation of covert attention [8]. In the present study we investigate shifts of the FoA elicited by external visual cues [1, 2, 5]. For instance, in the two-stripes test above one can direct the fly's attention to one side by briefly oscillating one of the stripes prior to the displacement. This increases the frequency of responses to the displacement of the stripe on the side of the oscillation and reduces responses to the stripe on the other side. The cue and the test can be separated in time as well as spatially [5].

In the first part of the present study we reinvestigate external visual cueing. It turns out to be behaviorally more complex than had been expected. In the second part we focus on the after-effect of cueing and show that it is mediated by dopamine signaling and that for this behavioral effect dopamine is required in the mushroom bodies (MBs).

The MBs are prominent symmetrical structures in the two brain hemispheres (e.g. [9]), each consisting of about 2000 intrinsic neurons, the so-called Kenyon cells. They have their dendrites dorso-posteriorly in the so-called calyx, their axons constitute the MB peduncle which anteriorly divides into the medial and vertical lobes. The mass of parallel Kenyon cells consists of 3 compartments, α/β, α’/β’ and γ. The α/β compartment is further subdivided into α/β_s_ (surface), α/β_c_ (core) and α/β_p_ (posterior). The α/β_p_ compartment can be distinguished from the main part of the MB by its morphology. Its about 90 Kenyon cells are connected to a special dendritic region, the ventral accessory calyx, separated from the main calyx, and they mix with fibers of other compartments in the β lobe[10]. The MBs are involved in a variety of complex brain functions such as learning and memory, sleep regulation, decision-making, context generalization and higher order motor control [11–15]. With the exception of learning and memory it is still obscure how the MBs contribute to these functions. We show here that interference with dopamine signaling in the α/β compartment and specifically the α/β_p_ Kenyon cells suppresses the after-effect of cueing.

## Materials and Methods

### Flies

Flies were cultured at 25°C on standard medium with 60% relative humidity on a 12h light/dark cycle. Wild type CantonS (CS) and GAL4 lines MB247, OK107 and c305 were of the Wuerzburg stock collection (Biozentrum, Department of Neurobiology and Genetics); c739 was obtained from Bloomington stock collection, NP1131 from Max-Planck-Institute of Neurobiology, Martinsried, Germany, c708 from Scott Waddell (Oxford University, UK). The mutant *radish*^*1*^ was provided by Josh Dubnau (Cold Spring Harbor Laboratory, Cold Spring Harbor, NY), the mutant *DopEcR* by Bertram Gerber (Leibniz Institute for Neurobiology, Magdeburg, Germany), the RNAi stocks were from VDRC stock center (#106961 and #12082; Vienna, Austria) and all *fumin* lines from Kazuhiko Kume (Nagoya University, Nagoya, Japan).

For tethering, we anesthetized 2 to 4 days old female flies by cooling and used a micromanipulator to glue (ESPE Sinfony™ DO3, 3M, Neuss, Germany) them to a triangular-shaped wire-hook made of copper (Ø = 0.05mm). This procedure also fixed the fly’s head to its thorax and prevented independent motion of the two body parts. After polymerization of the glue (10s pulse of blue LED light, < 0.5cm distance) flies were kept in single vials with access to water for a minimum of 2h.

### Pharmacological experiments

To influence dopamine signaling, 2 days old *radish*^*1*^ or CS flies were put for 14h on 5mg of Methylphenidate hydrochloride (Sigma) mixed under 10ml of regular food. To inhibit dDAT or DopR1 function, we kept 2 days old CS flies for 14h on 10ml of regular food with 30mg Desipramine hydrochloride or 1mg (+)-Butaclamol hydrochloride (Sigma), respectively. For inhibition of dopamine synthesis, freshly hatched CS flies were put on 20ml of regular food with 8mg α-Methyl-DL-tyrosine (Sigma) for 120h. We verified uptake of food by the addition of a non-hazardous blue dye, which after 14h stained the abdomen of the flies. To ablate the mushroom bodies, CS flies were treated with Hydroxyurea (Sigma) [16]. Dopamine was measured by HPLC (M. Krischke, Botany Department, University of Wuerzburg).

### Setup

**Fig 1:**
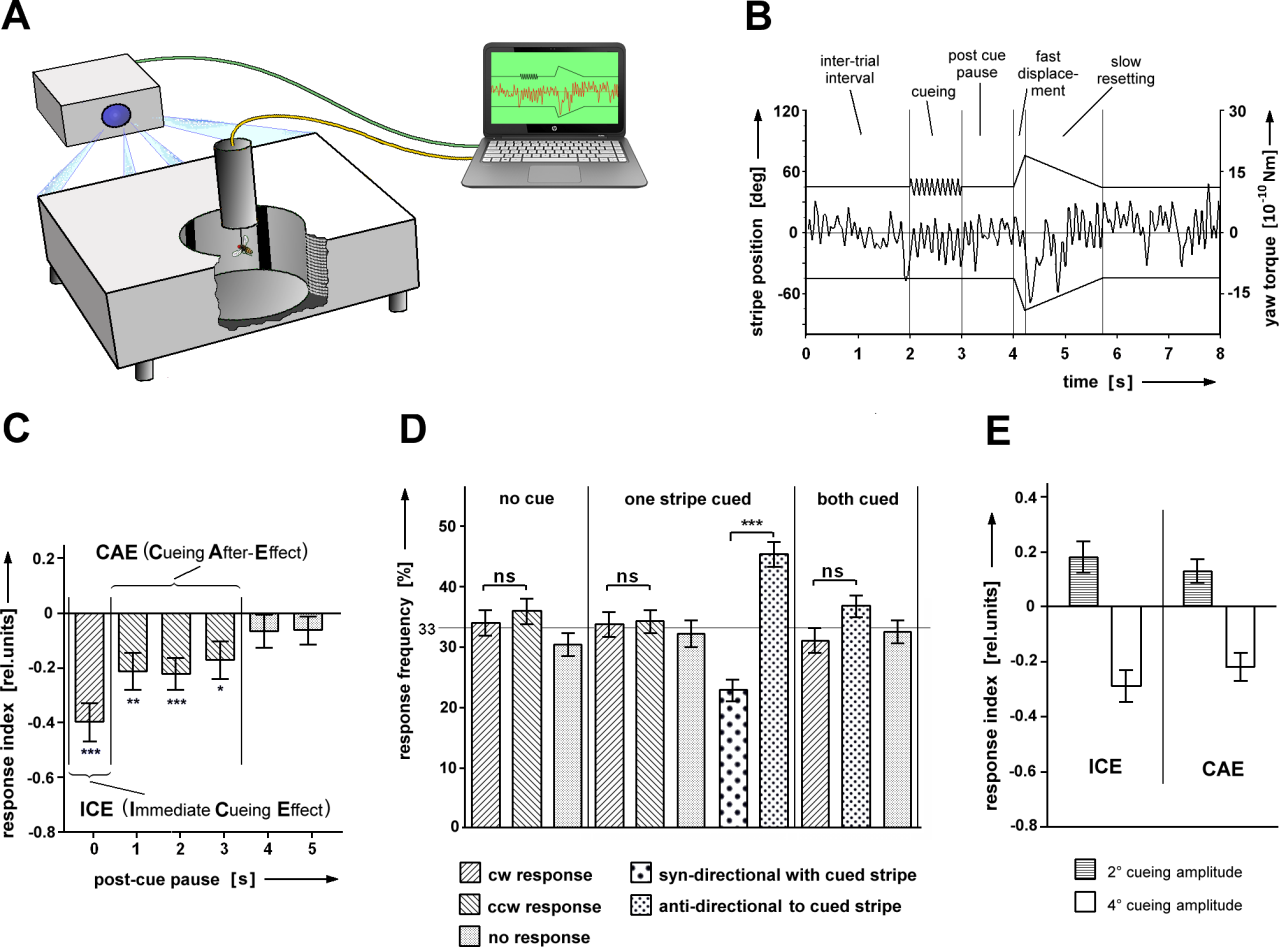
Cueing in selective visual attention. **(A)** Apparatus: Light-guide arena and torque meter. **(B)** Experimental procedure. The fly is attached to a torque-meter and positioned in the center of a light-guide arena. Stimuli are generated by a computer and projected onto one end of the light-guides. These display the visual stimuli at the inner surface of the arena. Two black stripes (width w = 18°; at ψ_0_ = + 45° and ψ_0_ = – 45°) are displaced from front to back on white background (Δψ = 30° at 150°/s) and then slowly reset to their initial position (v = 20°/s). They remain there for the inter-trial interval (ITI). The displacement may be preceded by an oscillation of one of the stripes (cue; e.g. Δψ_cue_ = 15°, f_cue_ = 10Hz and dur_cue_ = 1s) and a post-cue pause. **(C)** Immediate cueing and cueing after-effect. If the cue is immediately followed by the test, a strong bias in the response frequencies can be seen (ICE). It is slightly reduced, if a post-cue pause of 1, 2 or 3s is introduced (CAE). If the stripes remain stationary after the cue for 4 or 5s, the cue does no longer bias the responses towards the not cued stripe. Stripe width w = 18°; oscillation amplitude Δψ_cue_ = 15° (N = 52). **(D)** Cueing does not reduce the overall response rate. The same flies are tested either without cueing, with cueing of one stripe or cueing of both stripes (N = 71). **(E)** ICE and CAE are observed with positive and negative cueing (stripe width w = 6°; Δψ_cue_ = 2° for attractive cueing; Δψ_cue_ = 4° for repulsive cueing; N = 22). Error bars are SEMs (*P < 0.05, **P < 0.01, ***P < 0.001).

A fly was attached to the torque-meter and centered in a cylindrical arena (Ø = 90mm, h = 90mm) covering 360° x ±45° of the fly's visual sphere (Fig 1A). Visual stimuli were projected (BenQ W770ST, 120Hz) onto a rectangular plate, which held the ends of 180 (horizontal) x 32 (vertical) single light-guides. The other ends of the light-guides penetrated the wall of the arena, thereby transmitting the visual stimuli from the plate to the arena. The arena’s floor was covered with black cardboard and experiments were performed in a dark chamber. Position, timing and geometrical properties of the visual stimuli were controlled and updated at 300Hz using custom-made software (VB.NET). A torque-meter was used to measure the generated yaw-torque and the values were stored on the controlling computer’s hard disk at 100Hz. Experiments were performed under open-loop conditions, i.e. giving the fly no visual feedback of its generated yaw-torque.

### Stimulus conditions

Unless stated otherwise, two black 18° wide and ±45° high stripes were presented on a white background, centered at ψ_0_ = ±45° in the fronto-lateral visual field of the fly. The stripes were displaced from front to back by Δψ = 30° at 150°/s and then slowly reset to their initial position at 20°/s. The inter-trial interval was set to 2s and prior to each displacement a cue, followed by a post-cue pause was added. To cue, we oscillated one of the stripes (Δψ_cue_ = 15°, f_cue_ = 10Hz and dur_cue_ = 1s) and during the post-cue pause the stripes remained stationary. Usage of different cueing amplitudes and stripe width are stated in the corresponding text and figure legends. Six different sets of post-cue pauses (0, 1, 2, 3, 4 and 5s) were used in a typical experiment. A single set included six displacements, three times with cueing of the left stripe and three times of the right stripe. The order of sets as well as the order of the cued sides within each set was randomized. In experiments with a single stripe, both stripes were controlled by an identical protocol, but only one was shown/displaced. A response was scored when yaw-torque modulation exceeded the range between maximum and minimum yaw-torque values recorded within 0.5s prior to displacement by more than 60% within 0.5s after onset of the displacement. Left (counterclockwise; ccw) and right (clockwise; cw) responses as seen from the position of the fly were scored separately. If no sufficiently large yaw-torque modulation could be detected, a no response (nr) was scored.

### Data evaluation

Flies had to respond to at least 22 of the 36 test trials to be included in the evaluation. To quantify the effect of cueing, a response index (RI) was used to show the distribution of responses between the cued and the not cued stripe. It was calculated as RI = (rf_cued_—rf_uncued_) / (rf_cued_ + rf_uncued_) so that an equal number of responses towards and away from the cued stripe yielded a RI of 0. For every fly a separate RI was computed for each of the 6 different sets of post-cue pauses. All single fly RIs were then averaged for each post-cue pause. The wild type data consistently showed a tripartite pattern. The highest RI was found for post-cue pause 0s, whereas for pauses 4 and 5s the RI was not different from zero. Post-cue pauses 1-3s showed plateau values, which were significantly different from zero (Fig 1C). Thus, for averaging the data were grouped as post-cue pause 0s (immediate cueing effect, ICE) and post-cue pauses 1, 2 and 3s (cueing after-effect, CAE). In the experiments investigating the consequences of a reduced cueing duration or the efficacy of cueing in the upper or lower visual field, the CAE included only post-cue pauses of 1s.

### Statistical analysis

All data were tested for normal distribution using a Kolmogorov-Smirnov test. If data were normally distributed, a one-sample t test was used to compare values with zero and a two-sample t test was used to compare values with each other. Bonferroni corrections were used for multiple comparisons. If no normal distribution could be assumed, either a Wilcoxon Matched Pairs test for dependent pairwise comparisons or a Mann-Whitney test was used to test two groups against each other. A Wilcoxon-Signed-Rank test was used to compare values with zero. Comparison of more than two values was achieved by a one-way ANOVA with Holm-Sidak’s multiple comparisons test, if the data were normally distributed and otherwise with a Kruskal-Wallis test with Dunn’s test for multiple comparisons (* = p < 0.05, ** = p < 0.01, or *** = p < 0.001).

## Results

### A) Properties of visual cueing

#### A cue can be attractive or repellent

To study cueing in selective visual attention (SVA) we used the paradigm of [5]but with different stimulus parameters (see Material and Methods). In the two-stripes test stripes (w = 18°; ψ_0_ = + and– 45°) were simultaneously displaced front-to-back and reset slowly to their initial positions. Each of the two motion stimuli alone would have elicited a prevalently syn-directional response, incompatible with a simultaneous response to the other stripe. The simultaneous displacement forced the fly to choose one stripe and the fly tried to turn towards that stripe. To specifically address the effects of cueing on SVA, one of the two stripes was oscillated before the displacement (Fig 1B). A typical stimulus sequence consisted of an inter-trial interval of 2s during which the stripes remained stationary, followed by an oscillation of one of the stripes (Δψ_cue_ = 15°) for 1s and by a post-cue pause of 0-5s before the displacement and subsequent resetting of both stripes. As in the experiments of [5]the cue had a strong influence on the response frequencies, however, in the opposite direction. Responses towards the not-cued stripe became more, responses towards the cued stripe less frequent (Fig 1D). The overall response rate, as in the earlier experiments, remained constant. Even if both stripes were cued at the same time, the no-response rate stayed the same (Fig 1D). Thus, with the present experimental conditions in the light-guide arena the cue was repulsive (Fig 1D), while in the LED arena [5] it had been attractive. Apparently, a cueing event on one side prior to the test can make one of the targets more attractive or repellent than the other.

Moreover, the fly may just ignore the cue. [5]had observed that the flickering of a bright stripe of LEDs at the position of one of the test stripes had no cueing effect. As in previous studies [5, 6], in the present study the same cueing event was presented over and over again with one or the other stripe, up to 36 times. With none of the cueing conditions it was always attractive or always repellent. It merely influenced the ratio of the response frequencies towards and away from the cued side. As in a single series the cueing event was always the same, we assumed that also the evaluation of it by the fly was the same (attractive or repellent), but that the cueing was sometimes effective and sometimes ineffective. Alternatively, however, the result of the evaluation of the cueing event by the fly might have changed from trial to trial and the ratio of response frequencies just reflected the time ratio of assessments of the cueing effect as attractive and repellent by the fly. This alternative could not be ruled out, but we consider it unlikely.

We successfully reproduced the positive cueing in the LED arena with the old stimulus parameters. In those experiments the cue had been followed immediately by the test (post-cue pause = 0s). As mentioned above, with this time course (ICE: Immediate Cueing Effect) the cue in the new arena had a slightly larger effect than with the post-cue pause of 1-3s (cueing after-effect; CAE; Fig 1C).

Next, we checked various stimulus conditions in the new setup to see under which conditions cueing was attractive and under which repulsive. Reducing the luminance of the light-guide arena (127.0μW/cm^2^) to a value (4.0μW/cm^2^) lower than that of the LED arena (17.5μW/cm^2^) did not restore attractive cueing. With narrow test stripes (w = 6°) and an oscillation amplitude of Δψ_cue_ = 4°) as was used by [5]cueing again was repulsive. However, with a smaller oscillation amplitude (w = 6°; Δψ_cue_ = 2°) cueing was attractive. With the same flies cueing was attractive when the stripe was oscillated with a 2° amplitude and repulsive when the amplitude was increased to 4° (Fig 1E). With the 10Hz oscillation it was just the oscillation amplitude that mattered.

#### Selective visual attention in the light-guide arena

To gather further insights into SVA and cueing in the light-guide arena, we used broad stripes (w = 18°) and a large oscillation amplitude (Δψ_cue_ = 15°). Repulsive cueing was observed under various conditions, such as inverted contrast, with male flies instead of females, black stripes on green background, flicker instead of oscillation or just showing a different background color on one side for 1s as the cue. In two cases (flickering grey stripe and changing background color) only an ICE was observed but no CAE (S1 Fig).

**Fig 2:**
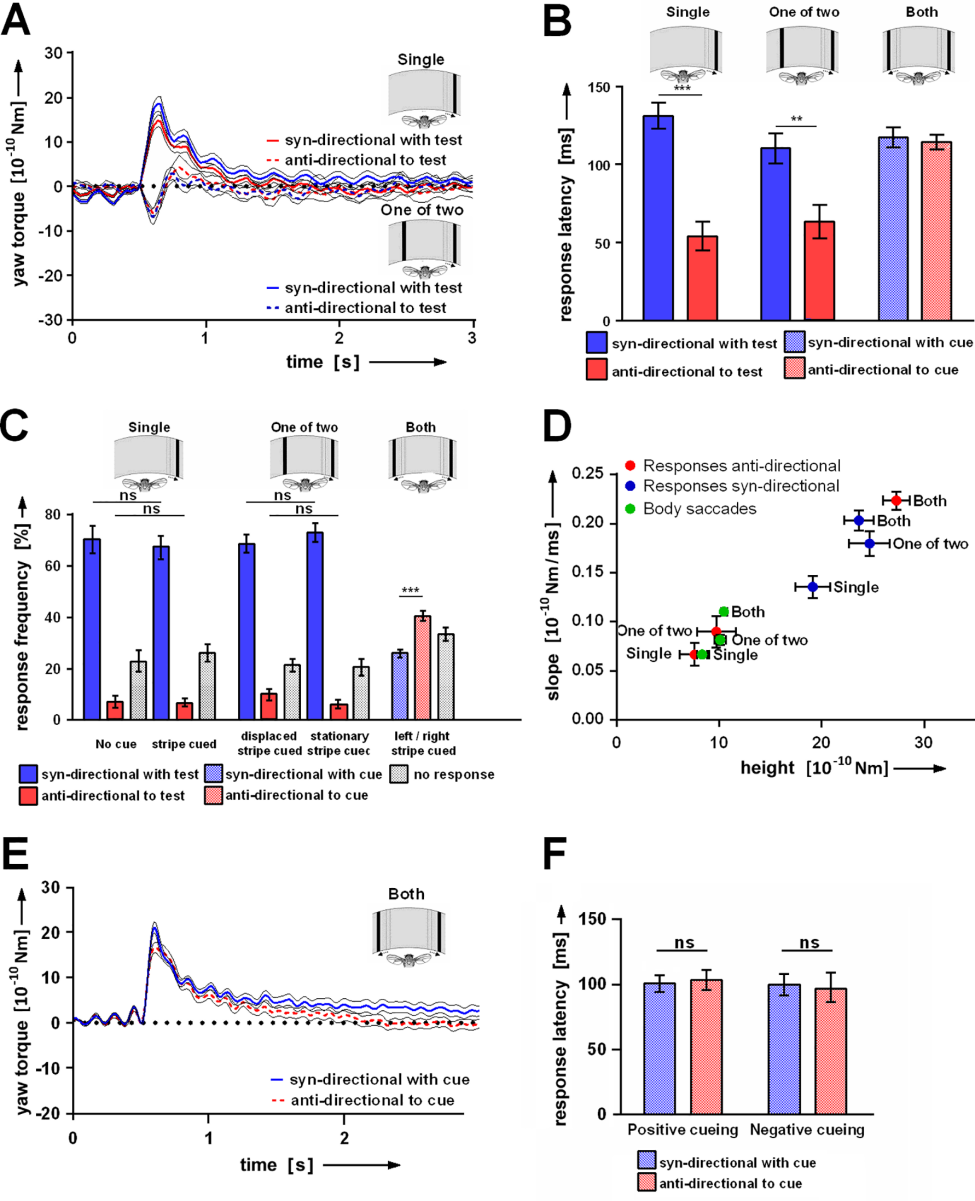
Characterization of yaw-torque responses. **(A)** Average yaw-torque traces of CS flies under two different experimental conditions. Flies respond to displacements of a single stripe syn- or anti-directionally. No differences can be seen in the responses with regard to the absence (‘Single’, N = 25) or presence (‘One of two’, N = 35) of a second stationary stripe. **(B)** Response latencies. Anti-directional responses to a single stripe are faster than syn-directional ones. No such fast responses can be observed, when two stripes are displaced. **(C)** Response frequencies. If only one stripe is displaced, the majority of responses is towards the direction of motion of this stripe. Only a small fraction of anti-directional responses can be observed. The simultaneous displacement of two stripes causes an increase in no responses and the cue biases the responses towards the not cued stripe (‘Both’, N = 52). **(D)** Slope and height of yaw-torque responses (red and blue) and spontaneous body saccades (green). Responses are grouped as syn- and anti-directional. No such distinction can be made in the ‘Both’ condition. Syn-directional responses differ in the analyzed parameters from anti-directional ones, which in turn are comparable to body saccades. **(E)** Average yaw-torque traces of CS flies in response to the simultaneous displacement of two stripes. Responses, syn- and anti-directional to cue, both resemble the syn-directional responses to a single stripe. **(F)** Response latencies have a similar value of about 100ms (N = 22), irrespective of whether the cue biases the response frequency towards the cued (‘Positive cueing’) or the not cued stripe (‘Negative cueing’), Error bars are SEMs (*P < 0.05, **P < 0.01, ***P < 0.001).

We went back to analyzing the effect of cueing in the response to the displacement of a single vertical black stripe (Fig 2). In half of the trials the stripe oscillated (cueing event) prior to the sudden shift. Most often the fly responded with a strong phasic yaw-torque modulation in the same direction as the motion stimulus (Fig 2A, Single, syn-directional) To show the time course of the yaw torque responses, these were synchronized for averaging at the beginning of the rising phase. Response latencies are shown in Fig 2B. If the fly would have been in closed-loop with its visual surround this response would have served to counter-balance the stimulus motion. Remarkably, in rare cases the fly generated yaw-torque spikes with opposite polarity to that of the displacement (Fig 2A, Single, anti-directional to test). Their latency was only about half the latency of syn-directional responses (Fig 2B, Single). They had a smaller amplitude and during the resetting of the stripe the fly generated a weak syn-directional response before its yaw-torque reached the base line again (Fig 2A, Single, anti-directional). The fast anti-directional responses might be interpreted as some kind of escape behavior. In ~25% of the tests the fly showed no response at all. The cue prior to the displacement did not influence the frequency of occurrence of the response types (Fig 2C, Single, no cue and stripe cued).

Similar yaw-torque patterns could be found, if the fly faced two stripes at ψ_0_ = + and– 45° and only one of them was displaced. The overall response frequencies remained the same and it again did not matter whether the displaced stripe was cued or not (Fig 2C, One-of-two, displaced stripe cued and stationary stripe cued). Also the response latencies of syn- and anti-directional responses were about the same in the ‘Single’ and ‘One-of-two’ conditions (Fig 2B).

In between trials the fly occasionally generated spontaneous yaw-torque spikes (body saccades, [2, 17]). Their response pattern resembled that of anti-directional responses and they could thus clearly be separated from syn-directional ones. As their dynamics nevertheless differed between the ‘Single’ and the ‘One of two’ experiments, it is likely that they were influenced by stimulus parameters other than the motion of the stripe (Fig 2D).

Interestingly, if both stripes were displaced, the fly increased the frequency of no responses (Fig 2C, Both) and suppressed the fast escape attempts (Fig 2B, Both and see below). Syn- and anti-directional responses with regard to the cued stripe looked much like the syn-directional (attractive) responses when only one stripe was displaced (Fig 2E). All these properties of SVA did not differ for repellent and attractive cueing if these were measured side by side in the same animals (w = 6°; Δψ_cue_ = 2 vs 4°; see for instance response latencies in Fig 2F).

#### Response polarity is independent of yaw-torque

**Fig 3:**
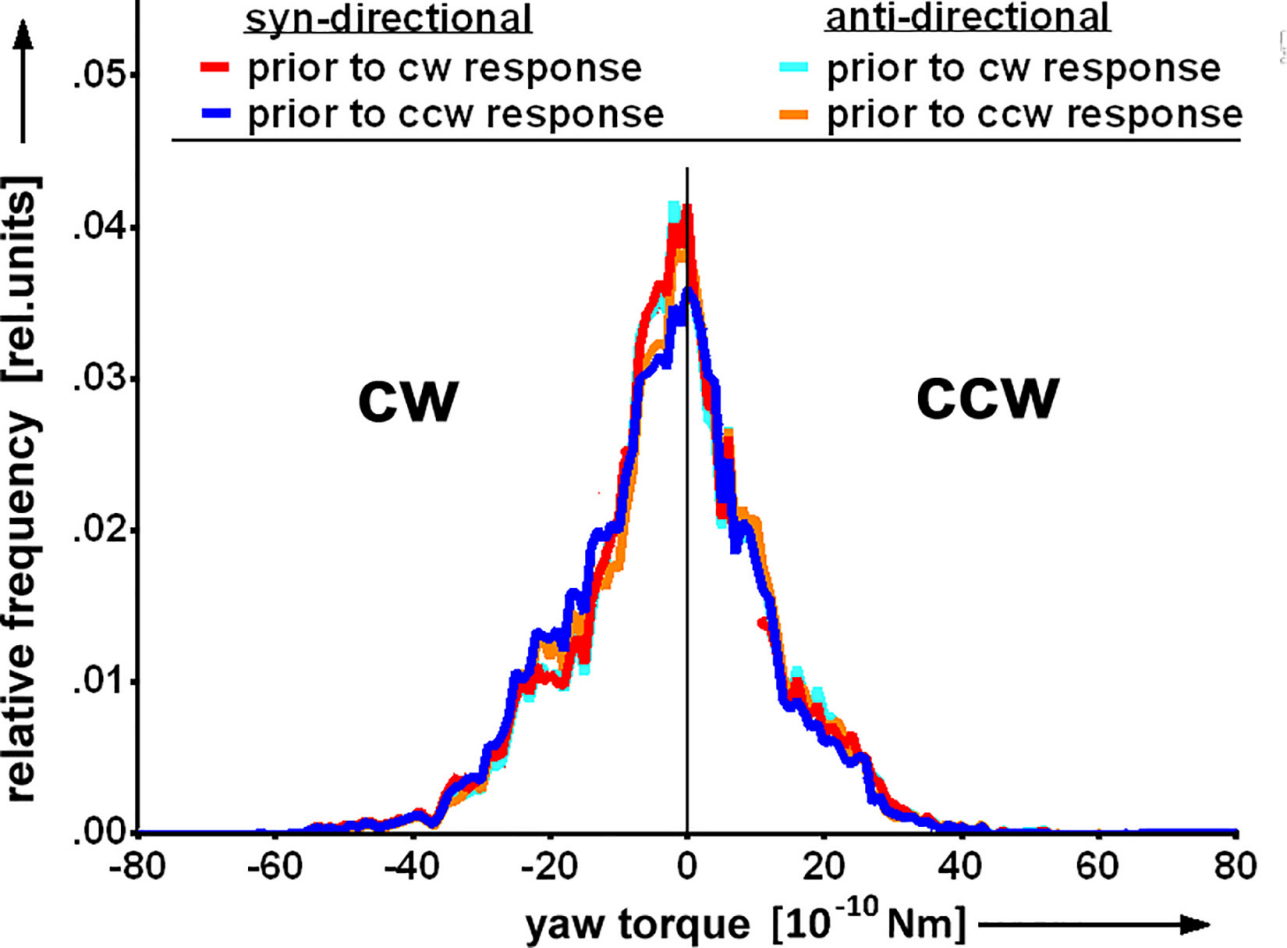
Yaw-torque level does not influence response polarity. Responses are grouped as (syn,cw_cue_), (anti,cw_cue_), (syn,ccw_cue_) and (anti,ccw_cue_). Histograms of CS yaw-torque values generated within 1s before the displacements show no difference (N = 138).

Using a visual learning task in the flight simulator, [3] provided evidence that yaw-torque and the location in the visual field to which a fly attends might be connected. Additionally, [4] found side-specific higher levels of brain activity depending on the direction in which a fly tried to turn. We looked for such a connection by analyzing the yaw-torque during 1s before the displacements and categorized it according to the cued side (cw_cue_; ccw_cue_) as well as syn- and anti-directional responses as ‘cw_cue_-syn’, ‘cw_cue_-anti’, ‘ccw_cue_-syn’ and ‘ccw_cue_-anti’. A putative influence of the preceding yaw-torque level on response polarity as well as an influence of the cue on the yaw-torque-level should thus become visible as a difference of the yaw-torque histograms of the 4 groups. No such difference was observed (Fig 3). The result again was the same for repulsive and attractive cueing (data for attractive cueing not shown). We conclude that the flies were able to shift their FoA independent of yaw-torque in our paradigm.

#### Visual field properties

**Fig 4:**
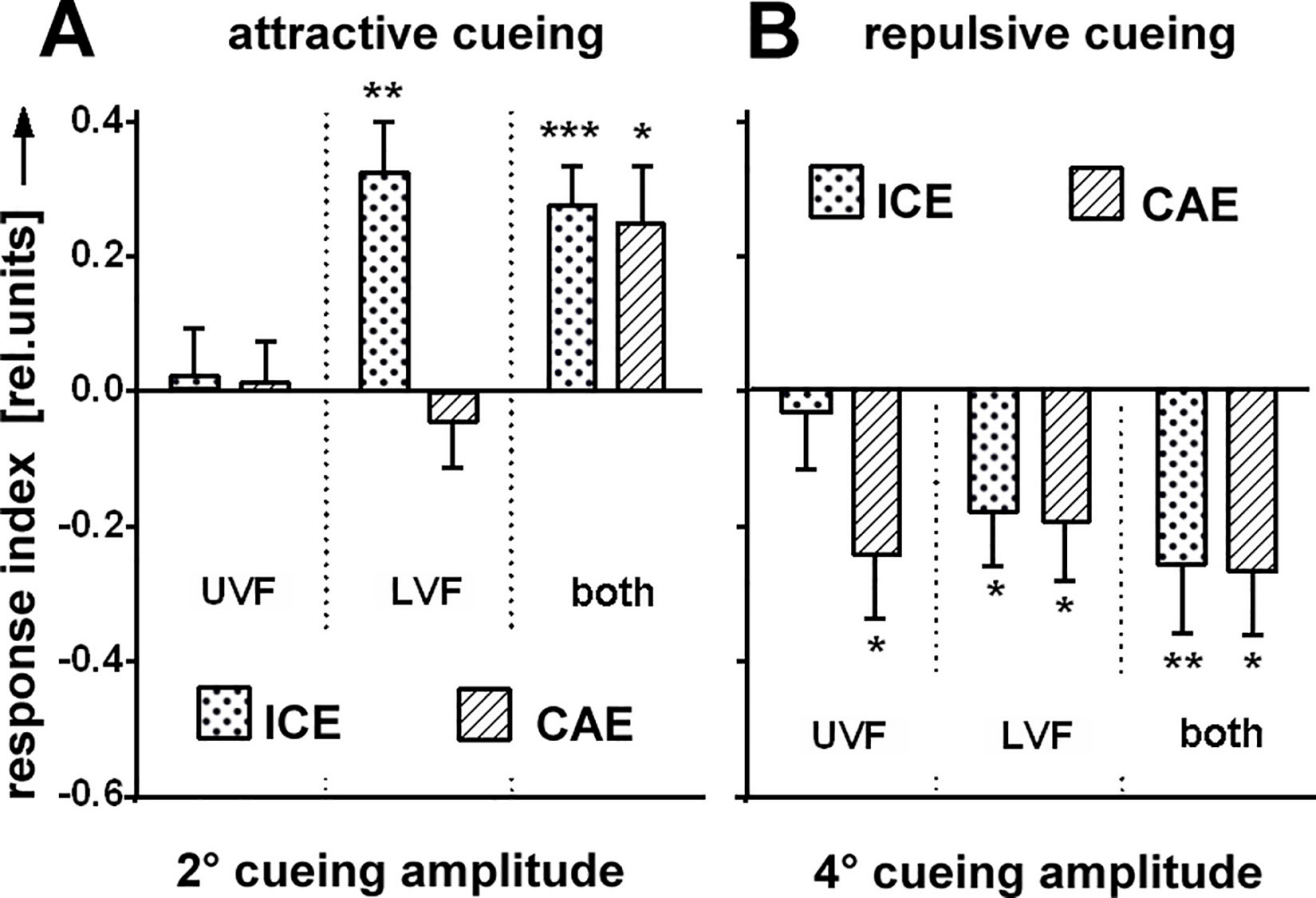
Visual field properties of cueing. **(A)** For positive cueing conditions (‘6°|2°’) no cueing is observed (N = 32) if the stripes are restricted to the upper visual field (UVF). If stripes are restricted to lower visual field (LVF) cueing elicits the ICE but not the CAE. **(B)** Negative cueing ('6°|4°’) elicits the CAE irrespective of whether the stripes are restricted to the UVF or LVF with the exception of the ICE which is elicited only in the LVF (N = 23).

[5] had discovered that the FoA could be cued in the lower but not in the upper visual field (LVF; UVF). In the light-guide arena we confirmed this effect for positive and negative cueing measuring the ICE. For the CAE, however, we found negative cueing in both the UVF and the LVF but positive cueing in neither (Fig 4A and 4B). For negative cueing in the UVF a CAE was observed but no ICE, implying that ICE and CAE might be independent of each other.

#### Is a repellent cue initially attractive?

**Fig 5:**
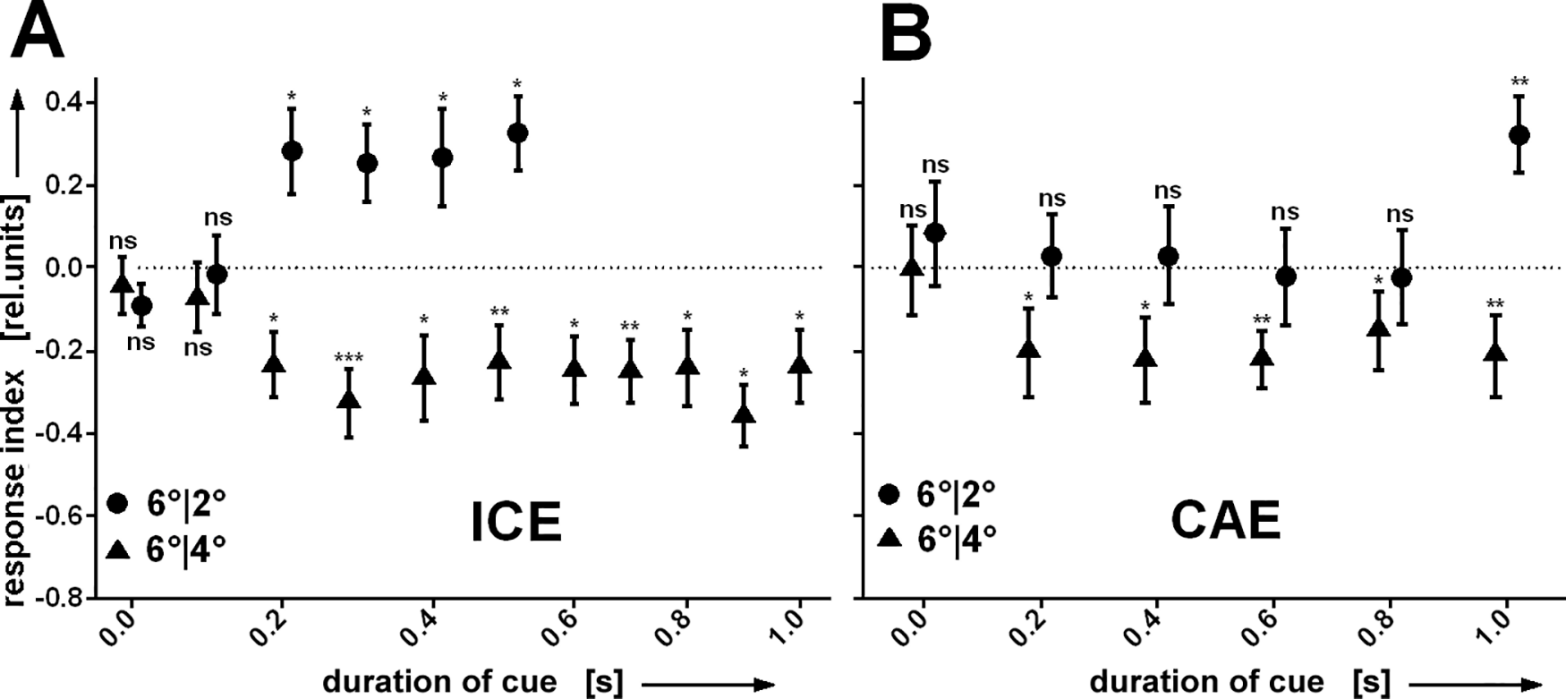
**(A) Reducing cueing duration.** If the cue duration is shorter than 0.2s no cueing is observed. Shortening of a negative cue ('6°|4°’, N = 23) does not make it less repellent. **(B)** For positive (‘6°|2°’, N = 22) but not negative cueing the oscillation has to last at least for 1s to elicit the CAE. All error bars are SEMs (*P < 0.05, **P < 0.01, ***P < 0.001).

We considered the possibility that repellent cueing might be a two-step process. The first appearance of the cue might attract the FoA and a short inspection of the cue might trigger the second step, a shift of the FoA away from this side. We therefore measured the dynamics of the cueing effect to see whether the repulsion was immediate or preceded by a short attractive phase. After a shortened cueing duration the FoA might still be on the cued side at the onset of the displacement. We shortened the cueing and measured both the ICE and the CAE. (The CAE in this experiment was measured with a post-cue pause of only 1s instead of 1-3s.) In short, no indication of an initially positive cueing effect was found (Fig 5A and 5B). At the earliest moment cueing could be observed it was negative. Cueing occurred already with two cycles of the oscillation (cueing duration = 0.2s), while 0.1s were not long enough to result in an ICE or CAE. This does not exclude the possibility that cueing might attract the FoA already with a short cueing duration, but this attraction would not show, since the response would not be activated before the FoA was shifted from the cued to the not cued side. For the ICE a similar picture emerged for positive cueing. The cue had to last for at least 0.2s to cause an ICE. Interestingly, no CAE was observed with positive cueing, if cueing was shorter than 1s. Again, what happened to the FoA before remains open.

### B) Neuronal mechanisms

#### Reducing dopamine via αMT abolishes the CAE

**Fig 6:**
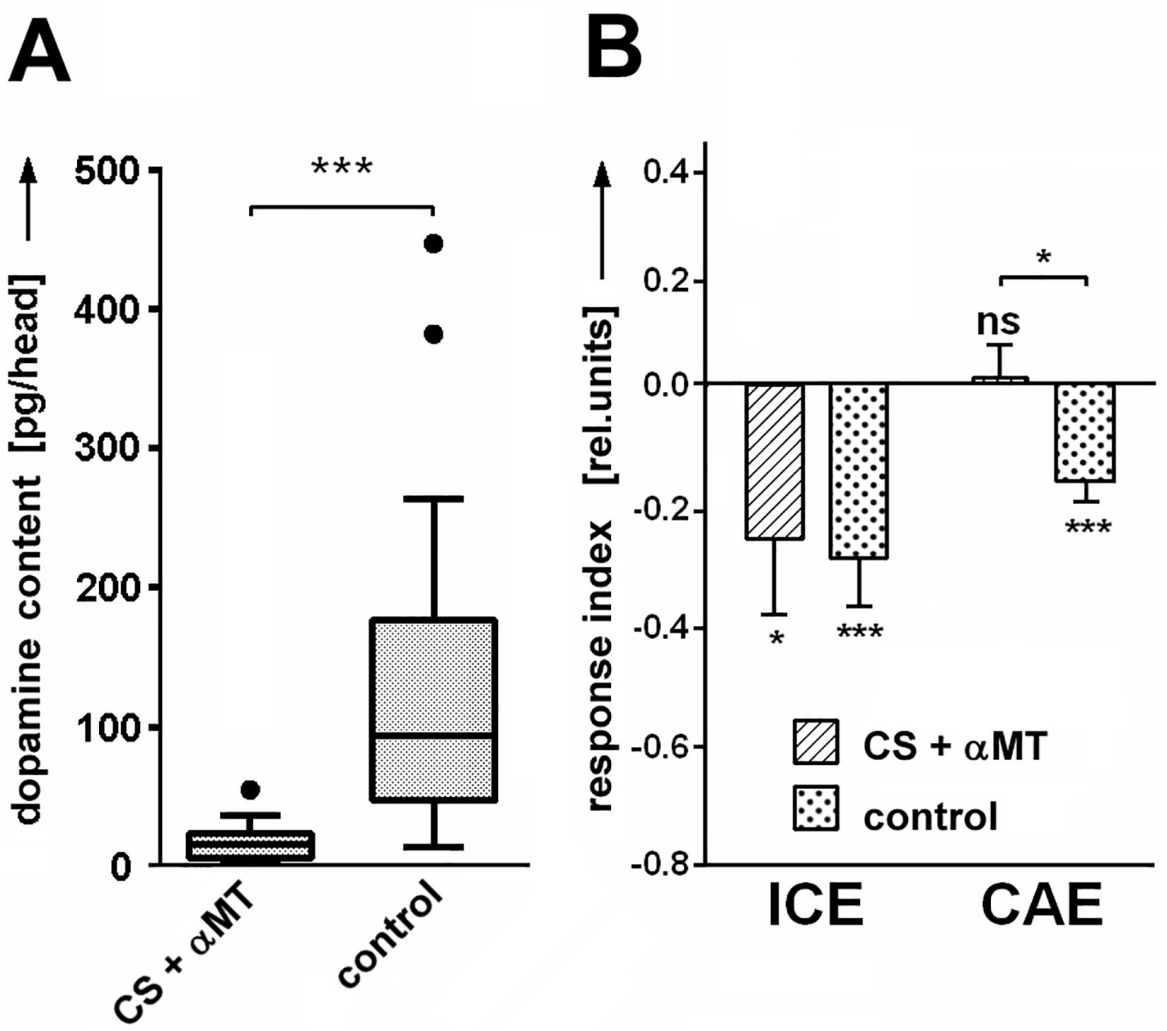
Reduced dopamine synthesis. **(A)** Feeding the drug α-methyl-DL-tyrosine (αMT) decreases dopamine levels in the fly [N (samples) = 19, 18; 20 heads per sample; Tukey whisker box plot]and **(B)** suppresses ICE and CAE [N (flies) = 23, 44; means and SEMs]. (*P < 0.05, **P < 0.01, ***P < 0.001). Dopamine concentrations in (A) are shown as boxplots with Tukey whiskers. If not otherwise stated, all N's are flies.

In many animal species attentional mechanisms have been shown to involve the dopaminergic system [18–23]. We wondered whether this also applies to SVA in *Drosophila*. We used negative cueing and recorded it immediately after the cue disappeared (ICE) and 1-3s later (cueing after-effect, CAE; Materials & Methods). Wild type flies were fed with α-methyl-DL-tyrosine (αMT), an inhibitor of tyrosin hydroxylase (TH), which in turn is a rate-limiting enzyme in the synthesis of dopamine (Fig 6A). The reduction in dopamine led to a loss of both the ICE and CAE (Fig 6B). Thus, sufficient amounts of dopamine are crucial for the cueing effect.

#### Interfering with the dopamine transporter

**Fig 7:**
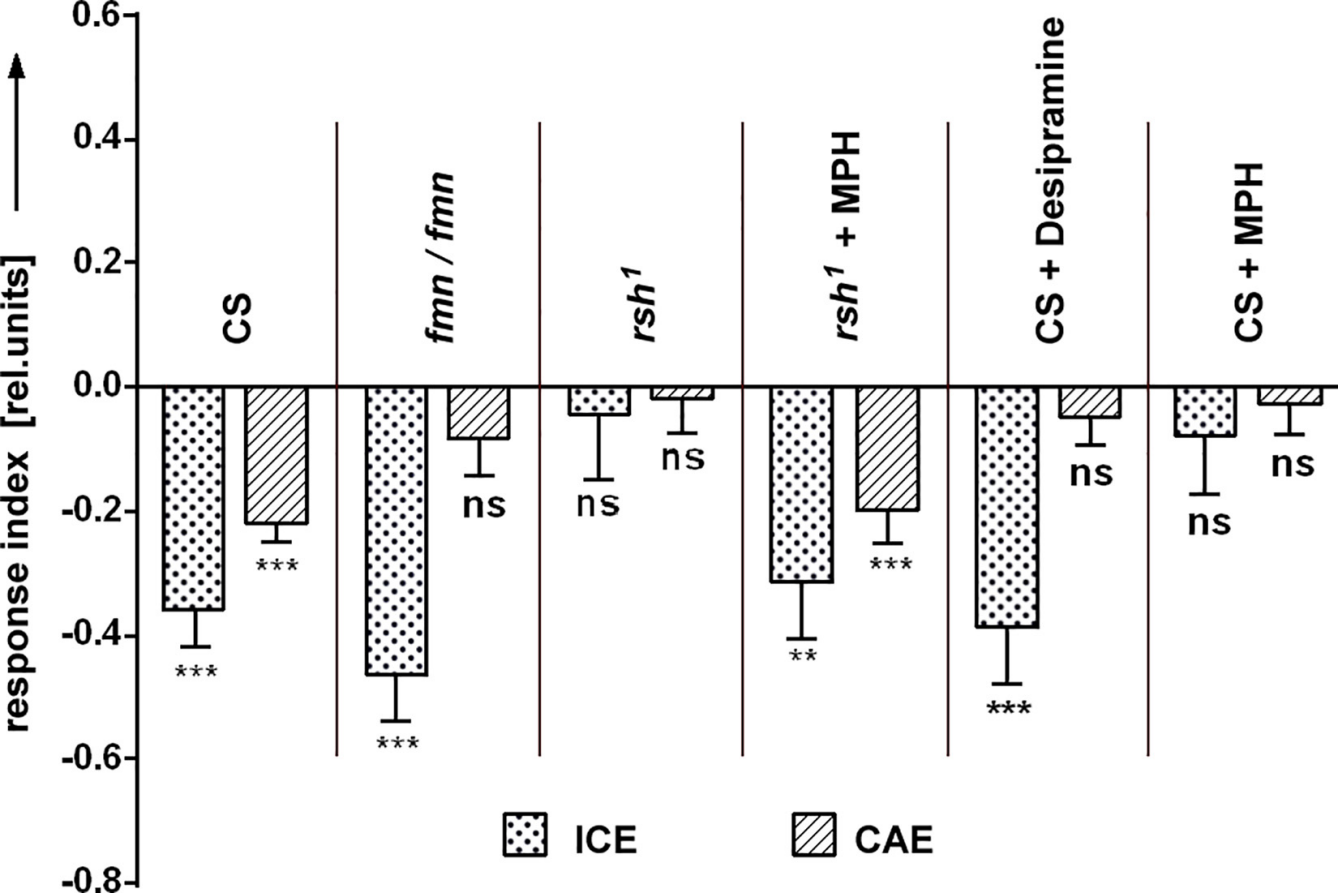
Dopamine re-uptake modulates the CAE. Flies with a lesion in the dDAT gene (*fmn*^*-*^*/fmn*^*-*^*)* still have an ICE but lack the CAE (N = 26). The same phenotype is found in flies heterozygous for this mutation. ICE and CAE are absent in *rsh*^*1*^ flies. Both defects can be rescued by inhibition of dopamine re-uptake via MPH (N = 21, 25). Pharmacological interference with dDAT function: Desipramine is an inhibitor of dDAT and its application leads to the dDAT^*fmn*^ phenotype (N = 26, 29). MPH is known to inhibit dopamine re-uptake via dDAT and causes a loss of CAE and ICE in wildtype CS (N = 41, 42). Error bars are SEMs (*P < 0.05, **P < 0.01, ***P < 0.001).

Mutant *fumin* (dDAT^*fmn*^ or *fmn*) flies have a defect in the gene for the dopamine transporter (dDAT), leading to a defective dopamine re-uptake and, in turn, to increased dopamine signaling [24] at dopamine synapses. dDAT^*fmn*^ flies show hyperactivity as well as alterations in their activity/rest pattern. Their yaw torque responses to stripe displacements were remarkably normal. We tested them for effects on cueing in SVA and found an intact ICE, but no CAE (Fig 7). Even heterozygous dDAT^*fmn*^/+ flies showed the same defects as the homozygous mutant animals (see (D) in last figure). As it appears, not only a decrease of intracellular dopamine but also an increase of extracellular dopamine at the synapse shortens the time course of cueing in SVA.

We confirmed the dDAT^*fmn*^ result pharmacologically, treating wild type flies with the drug Desipramine [24], an inhibitor of dDAT. Flies kept on food with 1mg/ml Desipramine for 14h resembled dDAT^*fmn*^ flies, showing intact ICE but no CAE (Fig 7). We also applied methylphenidate hydrochloride (MPH), a drug prescribed against attention deficit and hyperactivity disorder (ADHD). MPH targets the dopaminergic system and, in particular, the re-uptake of dopamine via dDAT [25]. Interestingly, MPH treatment of wild type blocked both ICE and CAE (Fig 7).

#### Mutant radish flies are impaired in cueing

A further gene of interest for the investigation of SVA is *radish* (*rsh*). It was initially found to play a role in memory and has later also been implicated in attention-like processes by [26]. Exploiting novelty choice behavior later described at the behavioral level by [27], they found a characteristic modulation of a local field potential in response to the novel stimulus. This particular modulation was less sustained in *rsh*^*1*^ flies, indicating a defect in short-term choice processes. We investigated cueing in mutant *rsh*^*1*^ flies. They showed neither an ICE nor a CAE while yaw-torque responses to test stimuli where normal. Studying attention-like behavior, [26] had presented evidence that feeding *rsh*^*1*^ flies with MPH rescued defects in selection/suppression dynamics of this behavior. In our study as well, the mutant phenotype in SVA could be reverted by feeding MPH to *rsh*^*1*^ flies (Fig 7). These flies behaved much like the wild type in terms of having a significant ICE, which was slightly higher than the also significant CAE. The result suggests that in *rsh*^*1*^ flies the impairment of cueing is due to a local shortage of dopamine at the relevant synapses, which can be compensated by MPH inhibiting dDAT.

#### Dopamine receptor DopR1 is involved in cueing SVA

**Fig 8:**
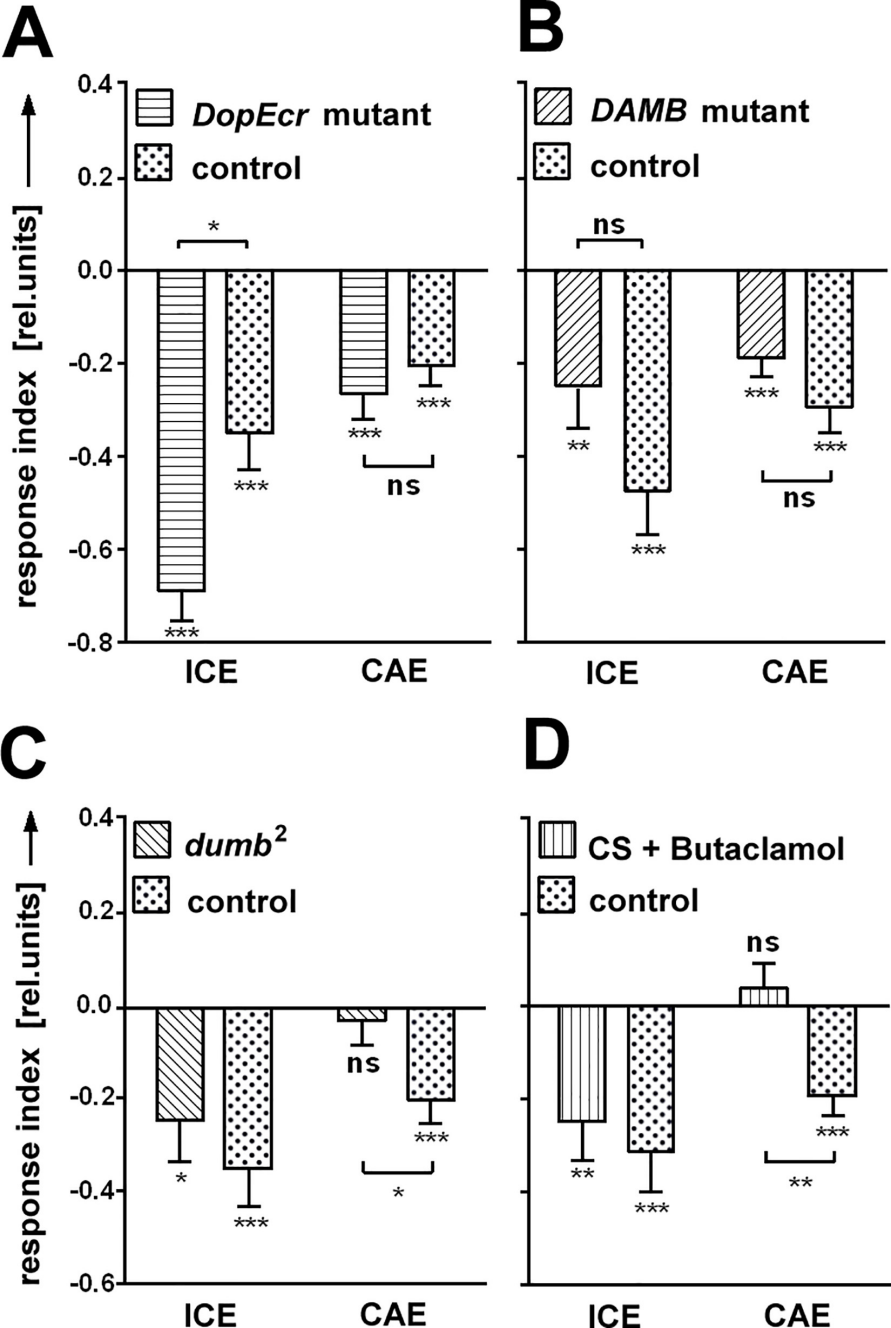
Dopamine receptors for SVA. **(A-C)** Out of three tested dopamine receptors only dDA1 (mutant *dumb*^*2*^*)* is critical for the cueing after-effect (N = 26, 35; N = 27, 30; N = 29, 35). **(D)** Pharmacological manipulation of dDA1 by the antagonist Butaclamol causes the same phenotype. Controls are CantonS (CS); mutants are homozygous, on CS background. All error bars are SEMs (*P < 0.05, **P < 0.01, ***P < 0.001).

To exert its effect as a neurotransmitter, dopamine needs to bind to a receptor. *Drosophila* has three types of dopamine receptors, two D1-like (DopR1, DopR2) and one D2-like (DD2R). They are involved in a wide range of behaviors such as learning, wakefulness, arousal and locomotion [28–31]. Additionally, in *Drosophila* there is a β-adrenergic-like G-protein coupled receptor called DopEcR with high expression in the CNS, which responds to dopamine as well as to ecdyson[32]. We obtained mutants for three of the four receptor genes to find out which of them was involved in SVA and particularly cueing. Flies without functional DopEcR were compromised neither in ICE nor in CAE. Cueing seems not to require DopEcR (Fig 8A). Likewise, DopR2 mutant flies (*DAMB*) showed no defects in SVA, displaying wild type like ICE and CAE (Fig 8B). The third mutant was *dumb*^*2*^, a hypomorphic DopR1 allele. We found a significant ICE, but no CAE in *dumb*^*2*^ flies (Fig 8C; for an expression pattern of DopR1 receptor see [29]). To independently confirm this result, wild type flies were put for 14h on food enriched with 1mg/ml Butaclamol, a DopR antagonist with higher affinity for DopR1 than for DopR2 [33–36]. The effects of this treatment resembled the phenotype of *dumb*^*2*^ mutant flies: a normal ICE, but no significant CAE (Fig 8D). Given that DopR2 mutant flies showed normal cueing, DopR1 seems to be the relevant target of Butaclamol. Taking all these results together the findings point at DopR1 as the most likely candidate for mediating the dopamine effects on the time course of SVA.

#### Mushroom bodies are required for cueing

**Fig 9:**
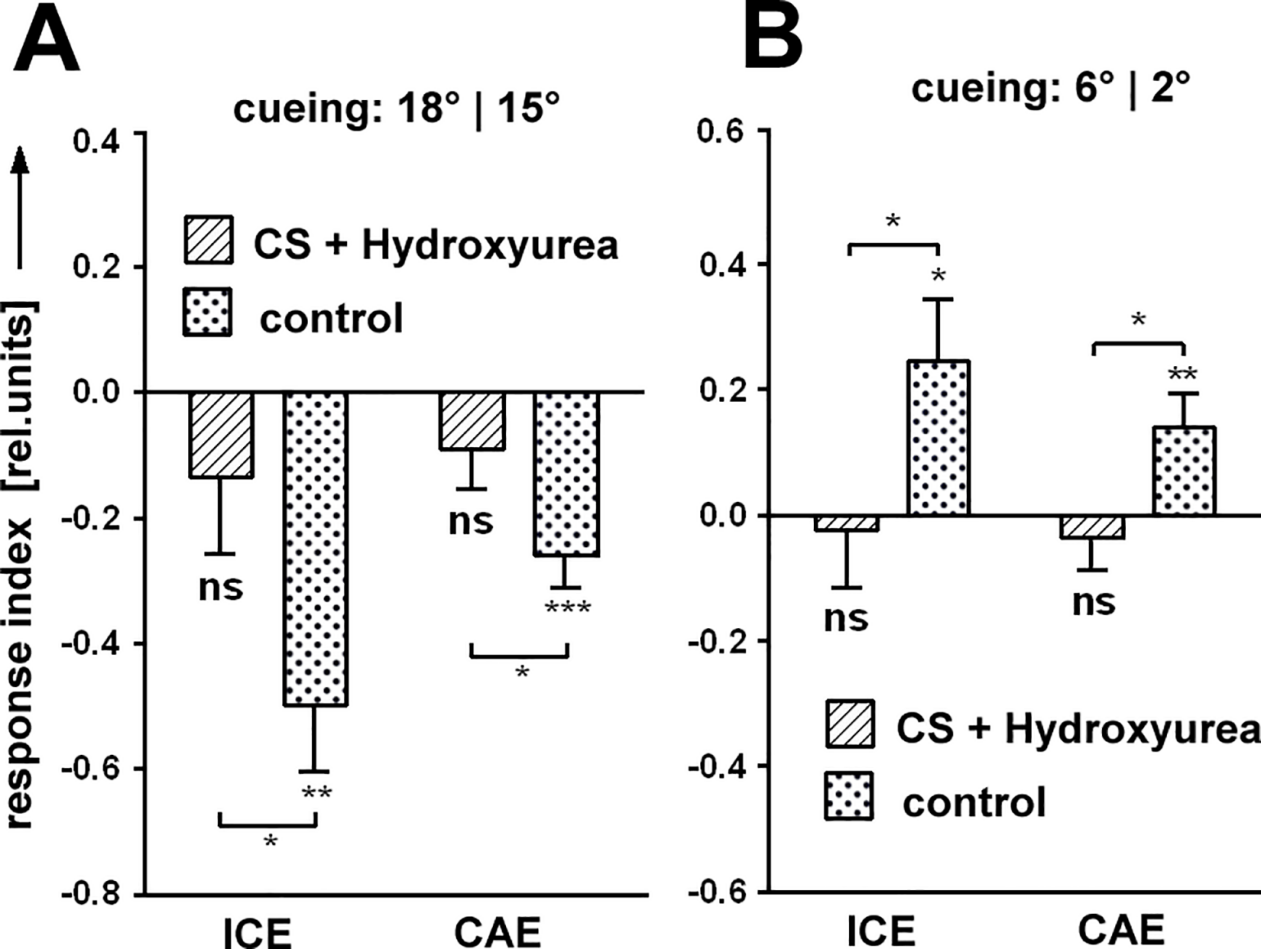
Mushroom bodies are required for cueing. After ablation of the MB neuroblasts in 1^st^ instar larva, adult flies show ICE and CAE neither in negative ('6°|4°’; N = 22, 24) nor positive cueing (‘6°|2°’; N = 29, 20), while the controls behave like wild type. Error bars are SEMs (*P < 0.05, **P < 0.01, ***P < 0.001).

As the dopamine system is intimately linked to the MBs and several of the genes used in this study are preferentially expressed there, we wondered whether the MBs are involved in SVA and specifically in cueing. We fed newly hatched larvae with hydroxyurea (HU, [16]), to ablate the MB neuroblasts and to obtain flies with MBs consisting of only embryonic KCs. We tested these flies and found normal test responses but neither a significant ICE, nor a CAE (Fig 9). This finding links SVA and in particular cueing to the MBs.

#### Only certain mushroom body compartments matter

So far we had shown that proper dopamine signaling, already 50% reduction of dDAT and the MBs each play a pivotal role in the CAE. Next, we probed the role of dDAT at the MBs as a combination of those three. Recently, a critical role of dDAT expression in the MBs has been reported for aversive odor memory as well as sleep regulation [37]. Here we use dDAT expression to assign the CAE to compartments of the MB. This analysis does not include the ICE since neither the transporter (Fig 7) nor the dDA1 receptor (Fig 8) appear to be required for it (ICE + CAE data shown as S2 Fig).

We used various MB Gal4-lines to express dDAT in a dDAT^*fmn*^/+ background in certain MB compartments. To test for necessity, we used RNAi against dDAT-mRNA in the wild type background to block the formation of dDAT protein exclusively in the Gal4-labeled cells. As dDAT is supposed to be a presynaptic dopamine transporter and Kenyon cells are not known to be dopaminergic, it is somewhat speculative to assume that wild type flies express dDAT in Kenyon cells. The results, however, suggest that this is the case.

**Fig 10:**
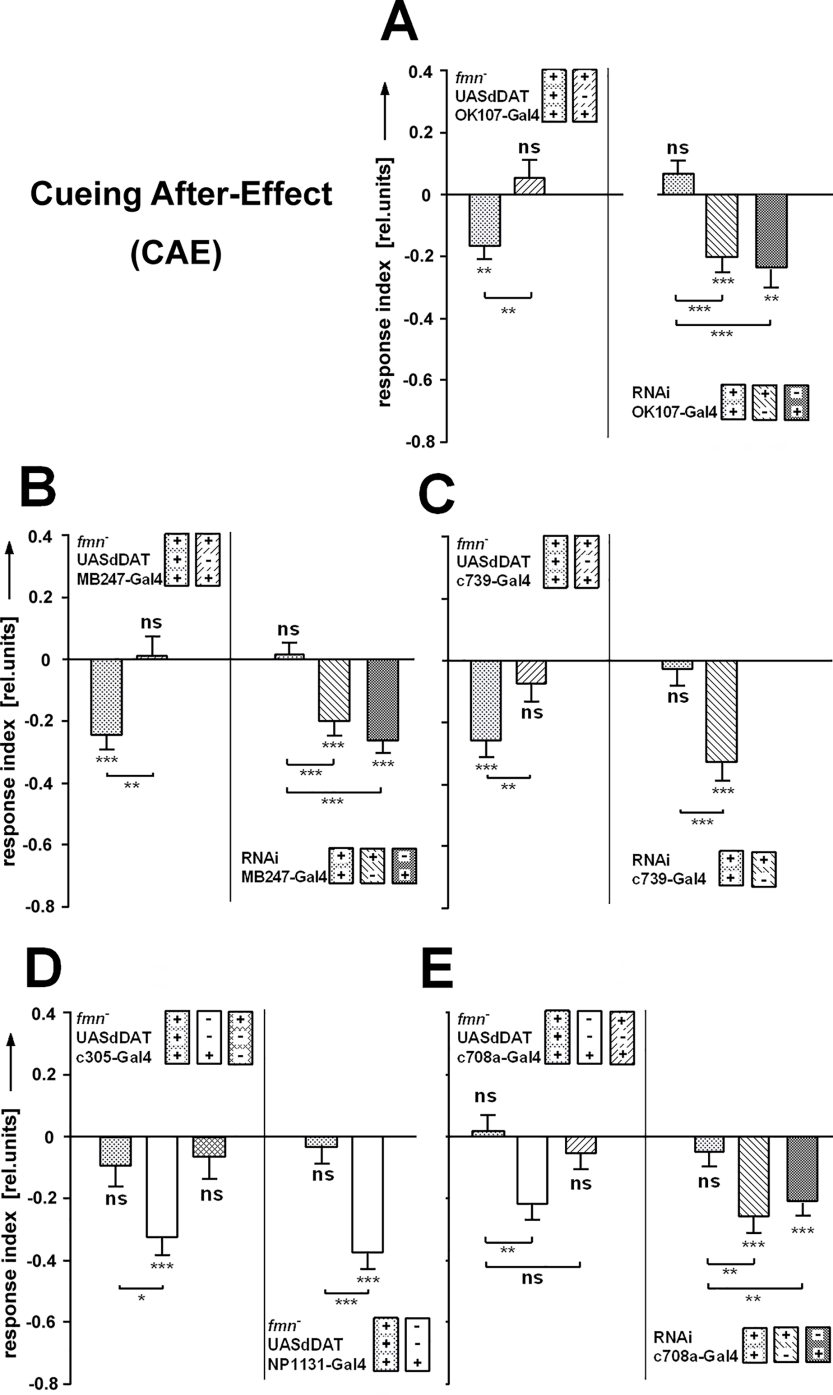
Rescue and knockdown of dDAT function in mushroom body compartments. **(A)** The OK107-Gal4 line labels all Kenyon cells (KCs) of the MBs and dDAT expression in these cells rescues the suppression of the CAE of the heterozygous *fmn*^*-*^ (dDAT^*fmn*^/+) flies (N = 38, 25). Knockdown of dDAT function in the same set of KCs leads to the absence of CAE, emphasizing the importance of the MBs for this behavior (N = 32, 29). **(B)** MB247-Gal4 has only marginal expression in the α’β’-lobes, but otherwise labels the same KCs as OK107-Gal4. Providing dDAT function in the labeled cells is sufficient to rescue the defect in CAE of the genetic control (N = 35, 17). RNAi against dDAT-mRNA in MB247-Gal4 labeled KCs leads to a dDAT^*fmn*^-like phenotype (N = 47, 29, 18). **(C)** c739-Gal4 driven expression of dDAT in the αβ-lobes is sufficient to rescue the dDAT^*fmn*^-like phenotype of the genetic control (N = 24, 27). Necessity of the αβ-lobes for CAE is demonstrated by the loss of CAE, if RNAi against dDAT-mRNA is expressed in the same set of cells (N = 27, 21). **(D)** c305a-Gal4 and NP1131-Gal4 label α’β’ and γ-lobes, respectively. Ectopic expression of dDAT in these cells in mutant dDAT^*fmn*^/+ background does not restore the CAE (N = 20, 25; N = 26, 19). **(E)** c708a-Gal4 has expression only in the αβ_p_ KCs. Also in these cells in dDAT^*fmn*^/+ flies dDAT expression does not restore the CAE (N = 29, 23, 20). Knocking down dDAT function in these cells, however, is sufficient to cause a dDAT^*fmn*^-like phenotype (N = 23, 21). All error bars are SEMs (*P < 0.05, **P < 0.01, ***P < 0.001).

We started with OK107-Gal4, which strongly labels the complete MBs. Restoring dDAT-function in all OK107-labeled cells of dDAT^*fmn*^/+ flies rescued the loss of CAE. Reciprocally, knocking down dDAT in those cells in wild type flies using RNAi suppressed the CAE (Fig 10A). Next, we drove dDAT-expression in a dDAT^*fmn*^/+ background with MB247-Gal4, a line that–like OK107-Gal4—labels α/β (including the accessory calyx) and γ-lobes, but has no (or only marginal) expression in α’/β’. The results showed a rescue of the CAE. Again, RNAi against dDAT-mRNA in the same set of cells removed the CAE (Fig 10B). In combination the results so far gave first evidence that α’/β’ might not be required for SVA. Next, we examined the requirement of the γ-lobe. Local dDAT-expression in the Kenyon cells of only the αβ-lobes (c739-Gal4) in a dDAT^*fmn*^/+ background restored the CAE and knocking down dDAT-function with RNAi in these same cells led to a dDAT^*fmn*^-like phenotype, proving αβ to be necessary and sufficient for CAE (Fig 10C). As additional controls, dDAT-expression driven in the α’β’-lobes by the driver line c305a-Gal4 and, respectively in the γ–lobes by NP1131-Gal4 (Fig 10D) failed to rescue the CAE. Taken together, dDAT-expression in αβ-, not in α’β’- or γ-lobes is necessary and sufficient for the after-effect of cueing in SVA.

The Gal4-line c708a has an expression pattern that specifically addresses the αβ_p_ KCs. A rescue of dDAT function in these cells was insufficient to restore wild type cueing in dDAT^*fmn*^/+ flies (Fig 10E). However, knocking down the dDAT protein in these 90 KCs in otherwise wild type flies caused a cueing defect (Fig 10E), ascribing a significant role for cueing in SVA to this small subset of cells. (Expression patterns of the above Gal4-lines (MB compartments) can be found in [9, 38].)

## Discussion

### Attractive and repulsive cueing

The unexpected finding that cueing is attractive, repulsive or ineffective reveals that also in stimulus-response situations the fly evaluates the stimulus according to the putative consequences of its response options [39]. Like all behavior, cueing in SVA takes part in natural selection. It is generated (or not) to secure survival and possible offspring.

### Properties of cueing in SVA

Having previously studied the attention span after endogenous shifts of the FoA in *Drosophila* [6], we now examined shifts of the FoA due to external cueing. The cue can be effective or ineffective, attractive or repulsive. It has no effect on the overall response frequency. It modulates the ratio of the response frequencies to the two stripes in relation to the cue. Sign and strength of the effect depend upon a variety of properties of the cue (e.g. visual field position, duration, salience, light intensity, S1 Fig). Repulsive responses after cueing are elicited with the same latency as attractive ones, require the same minimum cueing duration (for ICE) and in both there is no influence of the yaw-torque level prior to a response on the response polarity.

Evidently, the fly takes the cue not just as a marker for a particular location in the visual field. It evaluates in addition the cue's potential significance. In our study the fly is tethered and flying in a highly artificial experimental environment. The same cueing event occurs every few seconds. Even for these special experimental conditions our understanding of cueing is still rudimentary. Presumably, the special sensory properties of the cueing process we observe want to tell us something about the challenges of flight control under more natural conditions.

### Cueing after-effect and attention span

For the cueing after-effect (CAE) studied here and for the attention span in a previous study [6] the fly keeps the FoA for several seconds at a location to where it had shifted it. However, a cue is a signal from the outside world with a potential significance for the fly, whereas the attention span is a state maintained after the fly has actively shifted its FoA to a particular place. Still, similar processes at the neuronal or molecular level might underlie the two. For instance, both require a kind of working memory. Curiously, flies with a mutation in the *radish* gene showed a defect in both, the attention span and cueing. However, only the cueing is restored after feeding MPH (Fig 7 and [6]), indicating a different functional involvement of *radish* in the two processes.

### Are ICE and CAE the same process?

We would like to assume that the ICE and the CAE are just the same process recorded at different times after the cue. If so, however, we need to find an explanation for the result of Fig 4B showing with repellent cueing in the upper visual field a CAE but no ICE. Whether indeed the fly has a special mechanism for repellent cueing in the UVF, which suppresses or delays the cueing effect for a second, remains to be confirmed.

Genetic and pharmacological interventions in this study either interfered only with the CAE, or with both, CAE and ICE. Dopamine seemed to be mainly critical for the CAE while the ICE mostly remained undisturbed. In a few cases the manipulation of dopamine affected both, CAE and ICE. Two of them involved the mutant *rsh*^*-*^ and/or the drug MPH. Both are known to have other targets besides dopamine [21, 24]. As complete genetic removal of dDAT in the *fmn*^*-*^*/fmn*^*-*^ mutant (Fig 7) as well as reduced dopamine synthesis (Fig 6) leave the ICE intact one needs to search for other explanations for the suppression of the ICE in these cases. At present it has to remain open whether dopamine signaling is involved with cueing directly or just with its maintenance (CAE).

### SVA in mammals and the inverted-U hypothesis

Selective visual attention in mammals has long been known to involve dopamine signaling (e.g. [22, 40]). We now provide evidence for the involvement of dopamine in cued shifts of attention in *Drosophila*. It will be interesting to work out and compare the basic principles as well as the behavioral properties of visual attention in mammals and flies. For instance, dopamine signaling in mammals is described as a balanced process between too much or too little transmitter. It is called the inverted-U hypothesis [41] describing the relationship between task-performance and dopamine levels. In the present study the cueing after-effect (CAE) and sometimes also immediate cueing (ICE) vanished with increasing local dopamine concentrations caused by blocking its re-uptake and also with decreasing dopamine levels blocking its synthesis. Superficially, these findings seem to comply with the inverted-U hypothesis. However, in the fly brain the increase is only extracellular whereas the decrease due to blocked synthesis is primarily intracellular and presynaptic.

A further link between SVA in mammals and flies is the drug methylphenidate (MPH; commercial name: ritalin). [26] have argued that the altered short-term choice processes they found in the mutant *rsh*^*1*^ in a maze-walking paradigm might be a manifestation of impaired attention. Here we measured suppressed cueing of SVA in tethered flight in mutant *rsh*^*1*^ flies. The behavioral defects found in both paradigms could be rescued by feeding MPH, which is known to inhibit the re-uptake of dopamine from the synaptic cleft via dDAT [25, 26]. Once again, these findings suggest a parallel to attention in humans, where the drug is used to alleviate an attentional deficit (ADHD).

### The role of the αβp sub-compartment

Several independent evidences support the conclusion that for the CAE a significant level of dDAT is required in the Kenyon cells of the αβ compartment of the MBs and that dDAT matters only there for the CAE. Already a reduction of dDAT in the 90 Kenyon cells of the αβ_p_ compartment seems to be sufficient to suppress the CAE. How the αβ compartment and in particular the αβ_p_ Kenyon cells are involved in the CAE remains to be studied in detail. Interestingly, in optophysiological recordings [38] showed that the αβ_c_ and αβ_s_ Kenyon cells respond to odors by depolarization whereas αβ_p_ neurons are hyperpolarized by the same stimuli. This is in line with the special anatomy of αβ_p_. As mentioned in the Introduction olfactory input connections to the αβ_p_ fibers in the calyx are largely missing [10, 42]. Here for the first time we report a role of αβ_p_ in a visual task. Our data suggest that for the CAE the αβ_p_ sub-compartment is necessary but it might interact with one or both of the other two sub-compartments (αβ_c_; αβ_s_) as the restoration of dDAT in only the αβ_p_ neurons of the *fmn*^*-*^*/+* heterozygote was not sufficient to rescue the CAE. In the present study dDAT was manipulated in the postsynaptic cells. The level of dDAT in these neurons has been shown to affect DA signaling at, and dDA1 receptor concentration in these cells [37]. The transporter is supposed to keep the synaptic cleft in dopaminergic synapses free of dopamine and to limit the sphere of dopaminergic influence [43]. The above conclusions should be confirmed using the *dumb*^*2*^ mutant and GAL4 driver lines for all three sub-compartments (αβ_c_; αβ_s_; αβ_p_). Moreover, it should now be possible to identify the dopaminergic neurons innervating the relevant Kenyon cells and the pathway from the visual system to the MB [44, 45].

Selective visual attention equips visual systems of animals and humans with the fundamental ability to restrict their behavioral responses to stimuli in only part of their visual field. In flies one can now get at the underlying physiological and circuit mechanisms. Linking this process to dopamine in the αβ_p_ compartment of the MBs is a promising first step.

## Supporting Information

S1 Fig With many visual stimuli cueing is repulsive. Broad stripes (w = 18°) and a large oscillation amplitude (Δψ_cue_ = 15°). Repulsive cueing is observed with inverted contrast, with male flies instead of females, black stripes on green background, flicker instead of oscillations, 5s oscillations instead of 1s, or just showing a different background color on one side for 1s as the cue. Flickering a grey stripe and just changing background color on one side elicit only an ICE but no CAE.
(TIF)

S2 Fig Rescue and suppression of dDAT in mushroom body compartments has inconsistent effects on ICE. **(A)** OK107-Gal4 in heterozygous *fmn*^*-*^ (dDAT^*fmn*^/+) flies unexpectedly suppresses ICE. Additional dDAT expression in these cells rescues the suppression of the ICE (N = 38, 25). **(B)** Suppression of dDAT with RNAi in wild type in the same set of KCs leads to the absence of the ICE, emphasizing the importance of the MBs for this behavior (N = 32, 29). **(C)** MB247-Gal4 has only marginal expression in the α’β’-lobes. Otherwise the situation is the same as in **(A)**. Ectopic dDAT expression in these cells rescues the suppression of the ICE (N = 47, 29, 18). **(D)** Same as in **(B). (E)** c739-Gal4 in dDAT^*fmn*^/+ flies leaves ICE normal. Additional ectopic expression of dDAT in the αβ-lobes has no effect (N = 24, 27). **(F)** The necessity of the αβ-lobes for the ICE is demonstrated by the loss of ICE, if RNAi against dDAT-mRNA is expressed in the same set of cells in wild type (N = 27, 21). **(G—J)** dDAT modulation in c305-Gal4 (α’β’-lobes; N = 20, 25), NP1131-Gal4 (γ-lobes; N = 26, 19) and c708-Gal4 (αβ_p_ KCs; (N = 29, 23, 20; N = 23, 21) seems not to affect ICE. All error bars are SEMs (*P < 0.05, **P < 0.01, ***P < 0.001).
(TIF)
