# Supplementary figures and images for: Visual Attention in Flies—Dopamine in the Mushroom Bodies Mediates the After-Effect of Cueing
Source: PLoS One. 2016 Aug 29;11(8):e0161412. doi: 10.1371/journal.pone.0161412 (PMC5003349; doi:10.1371/journal.pone.0161412)

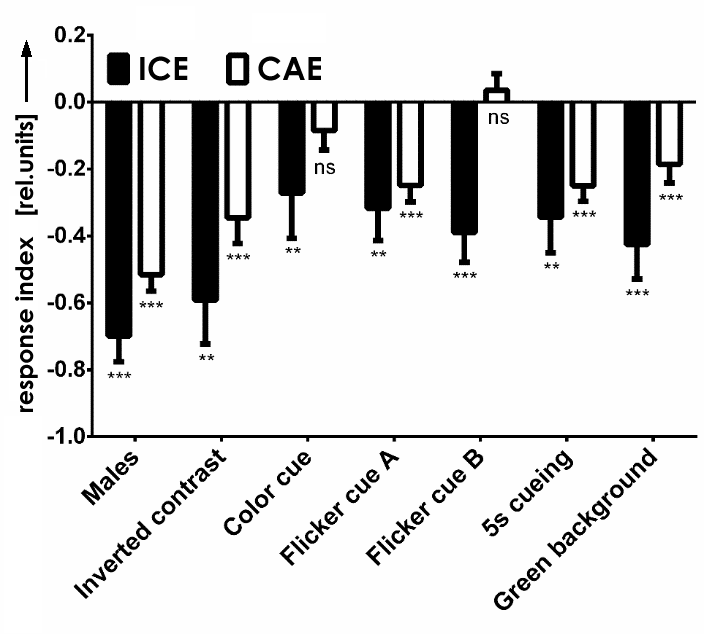

Supplement: S1 Fig — Broad stripes (w = 18°) and a large oscillation amplitude (Δψcue = 15°). Repulsive cueing is observed with inverted contrast, with male flies instead of females, black stripes on green background, flicker instead of oscillations, 5s oscillations instead of 1s, or just showing a different background color on one side for 1s as the cue. Flickering a grey stripe and just changing background color on one side elicit only an ICE but no CAE. (TIF) [file pone.0161412.s001.tif]

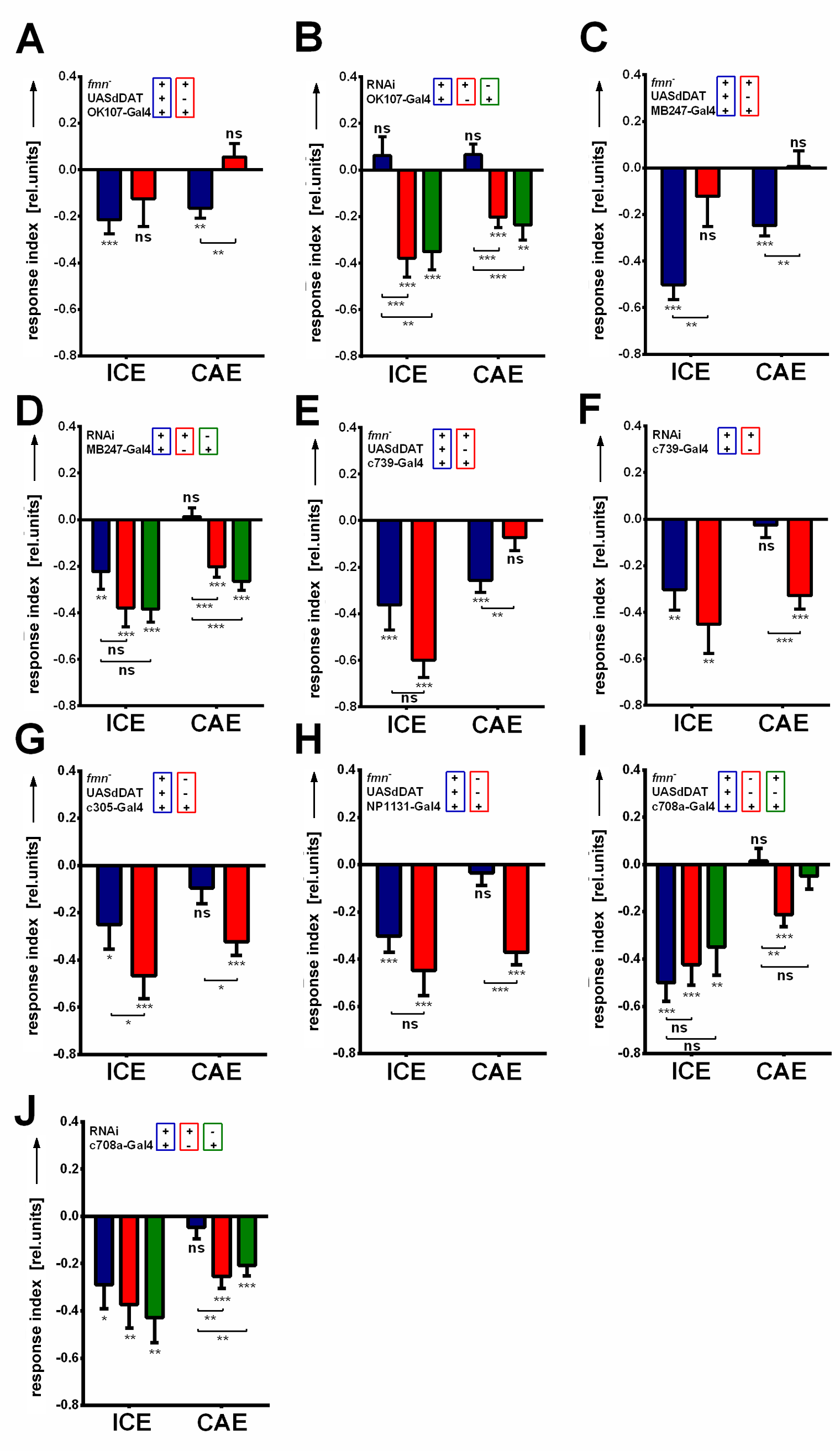

Supplement: S2 Fig — (A) OK107-Gal4 in heterozygous fmn- (dDATfmn/+) flies unexpectedly suppresses ICE. Additional dDAT expression in these cells rescues the suppression of the ICE (N = 38, 25). (B) Suppression of dDAT with RNAi in wild type in the same set of KCs leads to the absence of the ICE, emphasizing the importance of the MBs for this behavior (N = 32, 29). (C) MB247-Gal4 has only marginal expression in the α’β’-lobes. Otherwise the situation is the same as in (A). Ectopic dDAT expression in these cells rescues the suppression of the ICE (N = 47, 29, 18). (D) Same as in (B). (E) c739-Gal4 in dDATfmn/+ flies leaves ICE normal. Additional ectopic expression of dDAT in the αβ-lobes has no effect (N = 24, 27). (F) The necessity of the αβ-lobes for the ICE is demonstrated by the loss of ICE, if RNAi against dDAT-mRNA is expressed in the same set of cells in wild type (N = 27, 21). (G—J) dDAT modulation in c305-Gal4 (α’β’-lobes; N = 20, 25), NP1131-Gal4 (γ-lobes; N = 26, 19) and c708-Gal4 (αβp KCs; (N = 29, 23, 20; N = 23, 21) seems not to affect ICE. All error bars are SEMs (*P < 0.05, **P < 0.01, ***P < 0.001). (TIF) [file pone.0161412.s002.tif]
